# Block Adjustment without GCPs for Chinese Spaceborne SAR GF-3 Imagery

**DOI:** 10.3390/s18114023

**Published:** 2018-11-18

**Authors:** Guo Zhang, Qingwei Wu, Taoyang Wang, Ruishan Zhao, Mingjun Deng, Boyang Jiang, Xin Li, Huabin Wang, Yu Zhu, Fangting Li

**Affiliations:** 1 State Key Laboratory of Information Engineering in Surveying, Mapping and Remote Sensing, Wuhan University, Wuhan 430079, China; guozhang@whu.edu.cn (G.Z.); lifangting1985@163.com (F.L.); 2 School of Remote Sensing and Information Engineering, Wuhan University, Wuhan 430079, China; wuqingwei@whu.edu.cn (Q.W.); dmj2008@whu.edu.cn (M.D.); yuyinjby@163.com (B.J.); lixin_1995@whu.edu.cn (X.L.); 3 School of Geomatics, Liaoning Technical University, Fuxin 123000, China; zhaoruishan333@163.com; 4 Satellite Surveying and Mapping Application Center, Beijing 100048, China; whb@sasmac.cn; 5 Beijing Institute of Spacecraft System Engineering, Beijing 100094, China; zhuyubit@163.com

**Keywords:** GF-3, geometric calibration, RPC, without GCPs, planar block adjustment, accuracy

## Abstract

The Gaofen-3 (GF-3) satellite is the first C-band multi-polarization synthetic aperture radar (SAR) with the ability of high-accuracy mapping in China. However, the Ground Control Points (GCPs) are essential to ensure the accuracy of mapping for GF-3 SAR imagery at present. In this paper, we analyze the error sources that affect the geometric processing and propose a new block adjustment method without GCPs for GF-3 SAR imagery. Firstly, the geometric calibration of GF-3 image is carried out. Secondly, the rational polynomial coefficient (RPC) model is directly generated after the geometric calibration parameters compensation of each image. Finally, we solve the orientation parameters of the GF-3 images through DEM assisted planar block adjustment and conduct ortho-rectification. With two different imaging modes of GF-3 satellite, which include the QPSI and FS2, we carry out the block adjustment without GCPs. Experimental results of testing areas including Wuhan city and Hubei province in China show that the geometric mosaic accuracy and the absolute positioning accuracy of the orthophoto are better than one pixel, which has laid a good foundation for the application of GF-3 image in global high-accuracy mapping.

## 1. Introduction

Many spaceborne SAR systems in the world already have the ability of high-accuracy geometric positioning and mapping. The oldest SAR satellite is the European ERS satellite, and its plane positioning accuracy can reach 10 m [1]. The pixel location accuracy of the Sentinel-1A strip-map mode is at the sub-pixel level [2]. The COSMO-SkyMED satellite in Italy can achieve a geometric positioning accuracy of one m with the Spotlight-2 model [3]. For the TerraSAR-X satellite, the absolute positioning accuracy is 0.5 m in the azimuth direction and 0.3 m in the range direction [4]. The absolute location error (ALE) of Radarsat-2 is 17 m, which satisfies the system requirements of 40 m [5].

The GF-3 satellite is the first C-band and multi-polarization synthetic aperture radar (SAR) satellite in China [6]. The GF-3 satellite has 12 imaging modes. The resolution range is one to 500 m and the width is 10 to 650 km [7,8]. In order to verify the geometric accuracy achieved by the different imaging models of GF-3 images, Wang et al. analyze the SAR geometric error source and perform geometric correction tests based on the rational polynomial coefficient (RPC) model with and without ground control points (GCPs, playing a role in determining absolute datum) for the five imaging model. The root mean square (RMS) error of the independent checkpoints for the case of four corner control points is better than 1.5 pixels [9]. Ding et al. validated the geometric accuracy of the GF-3 SAR system by corner reflectors. The results show that the satellite positioning accuracy improved by three m [10]. The experimental results of Jiao et al. indicate that the proposed method can improve the geometric positioning accuracy of GF-3 images within two pixels [11]. For regional high-accuracy mapping, block adjustment of spaceborne SAR image is the key issue. Experts and scholars have conducted related research. Toutin determined the conditions of experimentation and application of path processing and block adjustment with SAR images when few controls were available [12]. Based on simultaneous multiple adjustments of critical SAR image parameters, Institut Cartogràfic de Catalunya presented a robust method to generate large-scale, high-quality digital elevation models (DEMs), using a set of SAR interferograms [13]. Spatiotriangulation is able to conduct simultaneous geometric processing of numerous images and strips, requiring only a few control points. Toutin evaluated its application to optical and SAR satellite images [14]. Wang et al. proposed planar block adjustment and orthorectification of Chinese spaceborne SAR YG-5 imagery based on rational polynomial coefficients [15]. Wang et al. prove that the proposed integrated orientation model can be effectively applied to the GF3 stereo pair. The GCPs, convergent angle, and weight setting have very important impacts on geometric accuracy [16]. In order to effectively solve the SAR ortho-photo problem caused by perspective contraction and overlay, a method is used by combining ascending and descending pass and DEM simulation to eliminate it [17,18].

However, through the above research, it is not difficult to find that block adjustment can only eliminate the inconsistency of positioning between images without GCPs, and there will exist system error in the overall absolute positioning accuracy. To improve the absolute positioning accuracy of SAR images, one approach is introducing GCPs in the adjustment process, but the acquisition of GCPs is usually very hard, especially in mountainous and unmanned areas. Another approach is the on-orbit geometric calibration of spaceborne SAR images. Zhao et al. used a multimode hybrid geometric calibration of spaceborne SAR, considering the atmospheric propagation delay, and all system errors can be effectively corrected through high-precision GCPs. The calibration results show that the system errors of GF-3 have been effectively eliminated, and the geometric positioning accuracy can be better than three m [19]. Deng’s study presents a geometric cross-calibration method for the GF-3 SAR system. They proposed a geometric calibration method without using corner reflectors and high-precision DEMs [20]. However, geometric calibration on the satellite mainly solve the problem of systematic error of a single image and is unable to take care of accidental error and mosaic accuracy of multiple images.

Therefore, considering the characteristic of geometric calibration and block adjustment, this paper combined the three steps effectively. First, the geometric calibration of GF-3 image is carried out and GCP is need in this part. Then, the rational polynomial coefficients (RPCs) are directly generated after the compensation of the geometric calibration parameters of each image. In this part, the main purpose is to simplify the subsequent processing, so using the RPC model to replace Range Doppler (RD) model. After replacing the model, many previous programs and software can be used to process both SAR and optical satellite images. Finally, the orientation parameters of the image are solved through DEM assisted planar block adjustment and conduct ortho-rectification. In this part, GCP is not needed at all. It is also the focus of this paper. The method proposed is expected to completely get rid of the dependence of ground control and achieve high-precision geometric positioning of spaceborne SAR image. At the same time, the relative mosaic and absolute positioning accuracy between images can be ensured. It brings a brand new process to the high precision block processing of SAR.

## 2. Principles and Methods

### 2.1. Geometric Calibration of Spaceborne SAR

Geometric Calibration of Spaceborne SAR is to find out and calculate the main error sources leading to geometric positioning system errors. Sensor instability, platform instability, signal propagation delay, terrain height, and processor error are the main factors affecting geometric positioning accuracy of spaceborne SAR [21]. For error characteristics, those that may affect the geometric positioning accuracy of spaceborne SAR can be classified as fixed system errors, time-varying system errors, and random errors, as described in the following subsections.

(1) Fixed System Error. The ranging signals of SAR system mainly depend on precise time measurement, including fast time (range direction) and slow time (azimuth direction). The two-dimensional time error is mainly affected by the time delay error of the SAR system and the azimuth time synchronization error, which is the main error source for the geometric positioning of spaceborne SAR. The radar signal through each component of the signal channel is the main cause of the time delay error. Time delay is mainly caused by the pulse-width and bandwidth of radar signal. The time delay errors of different pulse-width and bandwidth remain unchanged during SAR satellite operation. The error of time control unit of system equipment is the main factor leading to the azimuth time synchronization error. This error is relatively stable and does not change due to changes in the imaging modes for the same spaceborne SAR.

(2) Time-Varying System Error. Some of the error sources that affect geometric positioning accuracy are affected by time. These mainly include the atmospheric propagation delay error and the imaging processing error. The main factors affecting the atmospheric propagation delay of radar signals are atmospheric pressure intensity, temperature, water vapor content, ionospheric electron density, and the emission frequency of radar signals. Therefore, the atmospheric propagation delay error is a systematic error related to the incident angle of the radar beam and the imaging time of the SAR image.

(3) Random Error. In general, eliminating random error effectively by ground treatment methods is very difficult. Therefore, random error is the main factor affecting the theoretical limit of geometric positioning accuracy in the spaceborne SAR system. The random errors include predominantly satellite position error, SAR system delay random error, SAR antenna dispersion error, ground control point error, and atmospheric propagation delay correction model error [19].

In these error sources, the main error sources of spaceborne SAR are two-dimensional time errors, which mainly causes the geometric positioning error of SAR image in the range and azimuth direction. Thus, the geometric calibration model for spaceborne SAR is constructed as

(1) $$\{\begin{array}{c} t_{f}=\left(t_{f0}+t_{delay}+\Delta t_{f}\right)+\frac{x-1}{f_{s}}\\ t_{s}=\left(t_{s0}+\Delta t_{s}\right)+\frac{y-1}{f_{prf}} \end{array}$$

where *t_f_* and *t_s_* are, respectively, the fast time in range and slow time in azimuth; *t_f_*_0_ and *t_s_*_0_ are, respectively, the measured value of the starting time in range and azimuth; *t_delay_* is the atmospheric propagation delay time; Δ*t_f_* and Δ*t_s_* are the system delay time errors; *f_s_* is the sampling frequency, *f_prf_* is the pulse repetition frequency; and x and y are pixel coordinates.

The starting time of the satellite record is the time of radar signal received. The starting time in the azimuth direction will be affected by the imaging processing of GF-3 satellite. The intermediate time between transmitting and receiving time is the approximate equivalent SAR imaging time [22]. Therefore, it should be compensated for as:

(2) $$t_{s0}=t_{s0}^{\prime }-\frac{N/f_{prf}+t_{sample\_delay}}{2}$$

where $t_{s0}^{\prime }$ is the echo receiving time recorded on the satellite, *N* is the number of times the radar signal is transmitted from the transmitter to the receiver, and *t_sample_delay_* is the sample time delay in the satellite record.

The Range Doppler (RD) model is a rigorous geometric model for spaceborne SAR that establishes a rigorous relationship between the object space coordinate and the image space coordinate [19]. For the geometric calibration of SAR images, based on the RD model, earth ellipsoid equation and geometric calibration Equations (1) and (2), which is shown as Equation (3), N GCPs are used to calculate the geometric calibration parameters by using the least square method [23]. The GCPs are obtained from the corner reflector points or the central points of the cross road in the SAR image and are corrected according to the influence of solid earth tides (SET), which are calculated using the International Earth Rotation Service (IERS) Conventions 2003.

(3) $$\left\{ \begin{array}{l} R=\sqrt{{\left(X_{t}\;-\;X_{s}\right)}^{2}\;+\;{\left(Y_{t}\;-\;Y_{s}\right)}^{2}\;+\;{\left(Z_{t}\;-\;Z_{s}\right)}^{2}}\;=\;\left(t_{f0}\;+\;t_{delay}\;+\;\frac{x}{f_{s}}\right)\;\times \;c\\ f_{D}\;=\;-\;\frac{2}{\lambda R}\left(R_{s}\;-\;R_{t}\right)\;\times \;\left(V_{s}-V_{t}\right)\\ \frac{X_{t}^{2}\;+\;Y_{t}^{2}}{R_{e}^{2}}\;+\;\frac{Z_{t}^{2}}{R_{p}^{2}}\;=\;1 \end{array}\right.$$

where *R_s_* = [*X_s_ Y_x_ Z_s_*]^T^ and *V_s_* are the orbit vector; *R_t_* = [*X_t_ Y_t_ Z_t_*]^T^ and *V_t_* are the position vector and velocity vector of the target point; *f_D_* is the Doppler centroid frequency; *λ* is the radar wavelength; *R* is the slant range; *X* is the column number of the target point in the SAR image; *c* is the speed of light; $R_{e}$ is the mean equatorial radius; and $R_{p}\;=\;\left(1\;-\;1/f\right)R_{e}$ is the polar radius with a flattening factor of f = 298.255.

**Figure 1:**
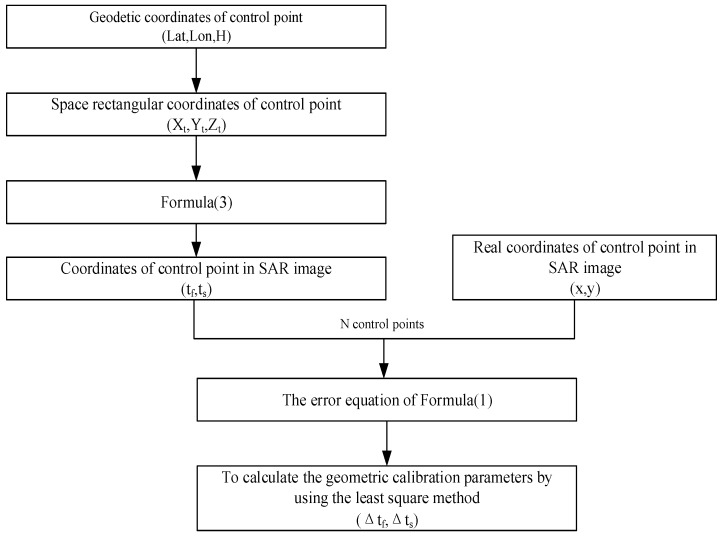
Flow chart of geometric calibration algorithm.

For the geometric calibration algorithm, based on the error equation of Equation (1), N control points are used to calculate the geometric calibration parameters by using the least square method, as shown in Figure 1. Then, the geometric calibration parameters are compensated to Equation (1). Based on the updated Equation (1), the geometric positioning accuracy after calibration is evaluated by Equation (3).

### 2.2. General Geometric Processing Model of Spaceborne SAR

Quite a lot of research teams have done the work of Block adjustment for optical imagery with RPCs. To facilitate the subsequent generalized and scaled processing, it is necessary to convert the compensated RD model parameters to the RPCs with terrain independent method. In this way, it is not necessary to model each satellite separately, and many optical imagery processing programs can also be directly used to process SAR images.

The related literature has shown that the RPC model is a generalized geometric model, which can be used to replace the RD model. The RPC model also establishes the relationship between the ground coordinates and the corresponding image coordinates. It makes full use of the auxiliary parameters of satellite images to create a general model, and can then be fitted to a RD model, and its fitting accuracy is better than 0.05 pixels [24]. In addition, in order to improve the numerical stability, we offset the 2D image coordinates and 3D ground coordinates, and scale it to the range of −1.0–1.0 by regularization parameter. The RPC model can be defined as follows [25]:

(4) $$r=\frac{Num_{L}\left(X,Y,Z\right)}{Den_{L}\left(X,Y,Z\right)}\;c=\frac{Num_{S}\left(X,Y,Z\right)}{Den_{S}\left(X,Y,Z\right)}$$

where X, Y, and Z are the normalized latitude, longitude, and height, respectively, L indicates the line, S indicates the sample, r is the normalized line number, c is the normalized sample number, and $Num_{L}\left(X,Y,Z\right)$, $Den_{L}\left(X,Y,Z\right)$, $Num_{S}\left(X,Y,Z\right)$, and $Den_{S}\left(X,Y,Z\right)$ are the terms of the third-order polynomial of (X,Y,Z).

For example, the form of the polynomial $Num_{L}\left(X,Y,Z\right)$ is as follows:

(5) $$\begin{array}{c} Num_{L}(X,Y,Z)={a_{i}}_{0}+{a_{i}}_{1}Z+{a_{i}}_{2}Y+{a_{i}}_{3}X+{a_{i}}_{4}ZY+{a_{i}}_{5}ZX+{a_{i}}_{6}YX+{a_{i}}_{7}Z^{2}+{a_{i}}_{8}Y^{2}+{a_{i}}_{9}X^{2}+{a_{i}}_{10}ZYX+\\ {a_{i}}_{11}Z^{2}Y+{a_{i}}_{12}Z^{2}X+{a_{i}}_{13}Y^{2}Z+{a_{i}}_{14}Y^{2}X+{a_{i}}_{15}ZX^{2}+{a_{i}}_{16}YX^{2}+{a_{i}}_{17}Z^{3}+{a_{i}}_{18}Y^{3}+{a_{i}}_{19}X^{3} \end{array}$$

where *a_ij_* (*i* = 1, 2, 3, 4; *j* = 0, 1, …, 19) are RPCs; there is a total of eighty parameters for RPCs.

The RPC model can be used as an alternative to the RD model, which is a traditional geometric SAR model. When the RD model is available, it is always possible to solve the parameters in a terrain-independent manner [24,25,26,27].

**Figure 2:**
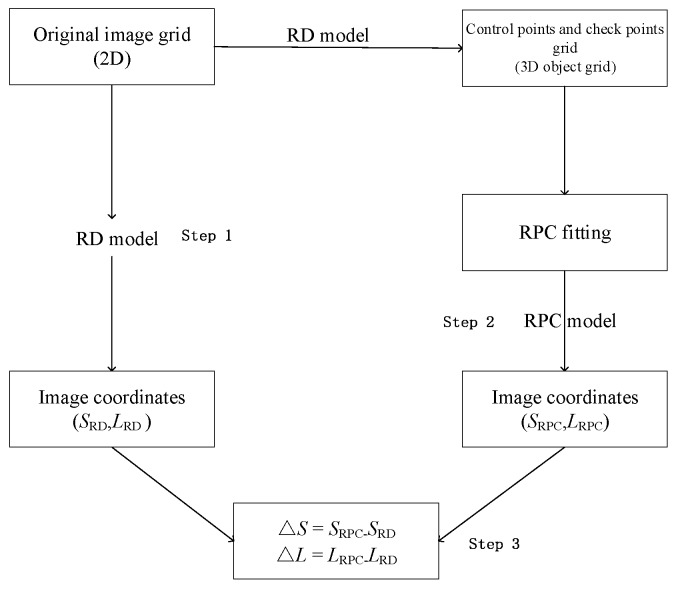
Flowchart of RPC model solution process.

The proposed estimation process, using a least-squares approach, requires only the RD model and the maximum and minimum heights in the image area, which can be extracted from the global DEM supplied by the United States Geological Survey. As shown in Figure 2, this method involves three main steps:

(1) Determination of an image grid and establishment of a 3D object grid of points using the RD model;
(2) RPC fitting; and
(3) Accuracy checking.

Experiments with different kinds of SAR data were carried out to verify this method, which show that RPC was able to replace the RD model [24].

### 2.3. Planar Block Adjustment Based on RPC

The RPC adjustment model uses an affine transformation to represent these two categories of difference between the calculated and the measured image-space coordinates. Similar to the traditional RPC-based block adjustment, the planar block adjustment does not correct RPCs, but merely corrects their affine transformation parameters, which is defined as follows [24]:

(6) $$\{\begin{array}{l} \Delta r=e_{0}+e_{1}r+e_{2}c\\ \Delta c=f_{0}+f_{1}r+f_{2}c \end{array}$$

where $\left(e_{0},e_{1},e_{2},f_{0},f_{1},f_{2}\right)$ are the affine transformation parameters and (Δr,Δc) are values used to compensate for systematic errors of the image point. The errors that can be eliminated by the relevant parameters have been introduced in detail in document [24].

Using Equations (5) and (6), the affine transformation parameters $\left(e_{0},e_{1},e_{2}\right)$ and $\left(f_{0},f_{1},f_{2}\right)$ of the image space compensation can be set as unknowns and be solved together with the plane coordinates X and Y of the ground point. Additionally, the area covered SAR images are often encountered the weak convergence geometric problem, which cannot be solved by traditional block adjustment, so a planar block adjustment based on RPC is carried out [15]. The elevation coordinates of Tie Points (TPs, playing as the corresponding points between images) are obtained by interpolating a DEM of the area, which primarily serves as a height constraint [28]. This method has been improved and validated in different test areas with Ziyuan-3 (ZY-3) optical satellite images [29]. The planar block adjustment error equation based on the RPC model is as follows:

(7) $$\left[\begin{array}{c} v_{r}\\ v_{c} \end{array}\right]=\left[\begin{array}{cccccccc} \frac{\partial r}{\partial e_{0}} & \frac{\partial r}{\partial e_{1}} & \frac{\partial r}{\partial e_{2}} & 0 & 0 & 0 & \frac{\partial r}{\partial X} & \frac{\partial r}{\partial Y}\\ 0 & 0 & 0 & \frac{\partial c}{\partial f_{0}} & \frac{\partial c}{\partial f_{1}} & \frac{\partial c}{\partial f_{2}} & \frac{\partial c}{\partial X} & \frac{\partial c}{\partial Y} \end{array}\right]\cdot \left[\begin{array}{c} \Delta e_{0}\\ \Delta e_{1}\\ \Delta e_{2}\\ \Delta f_{0}\\ \Delta f_{1}\\ \Delta f_{2}\\ \Delta X\\ \Delta Y \end{array}\right]-\left[\begin{array}{c} r-\hat{r}\\ c-\hat{c} \end{array}\right]\;p$$

where *v* is the residual vector of the image coordinate observation. *r* and *c* are the coordinates of the image point as measured manually; $\hat{r}$ and $\hat{c}$ are the coordinates of the image point as calculated by the RPC and affine transformation parameters; Δ*e*_0_, Δ*e*_1_, Δ*e*_2_, Δ*f*_0_, Δ*f*_1_, and Δ*f*_2_ are corrections of affine transformation parameters; Δ*X* and Δ*Y* are plane vectors containing increments of the ground point; and *p* is the weight of the observation equation. Due to the observation of equal weight, the value of *p* is 1. Equation (7) can be written in the matrix form:

(8) V=At+Bx−l   P

where V is the residual vector of the image coordinate observation, $t={\left[\Delta e_{0}\;\Delta e_{1}\;\Delta e_{2}\;{\Delta f}_{0}\;\Delta f_{1}\;\Delta f_{2}\right]}^{T}$ is the incremental vector of the affine transformation parameters, $x={\left[\begin{array}{cc} \Delta X & \Delta Y \end{array}\right]}^{T}$ is the incremental vector of the object space coordinates of the target point, A and B are coefficient matrices containing partial derivatives of the unknowns, and $l={\left[\begin{array}{cc} r-\hat{r} & c-\hat{c} \end{array}\right]}^{T}$ is the discrepancy vector. P is the unit matrix. As all the coordinates of image points are observations of equal precision, the initial value of P is an identity matrix.

The normal equation can be established from Equation (8) according to the principle of least-squares adjustment:

(9) $$\left[\begin{array}{cc} A^{T}PA & A^{T}PB\\ B^{T}PA & B^{T}PB \end{array}\right]\left[\begin{array}{c} t\\ x \end{array}\right]=\left[\begin{array}{c} A^{T}Pl\\ B^{T}Pl \end{array}\right]$$

After each adjustment, the plane coordinates of a tie point (TP) in the object space were refreshed, and an auxiliary DEM was used as the height constraint. Elevation *Z* in Equation (4) of the TP was interpolated from the DEM instead of the intersection of multiple SAR images. Along with the plane coordinates *X* and *Y*, *Z* was set as a new ground coordinate value of the TP and was subsequently substituted into the adjustment system for the next iterative calculation until the entire adjustment process converged.

**Figure 3:**
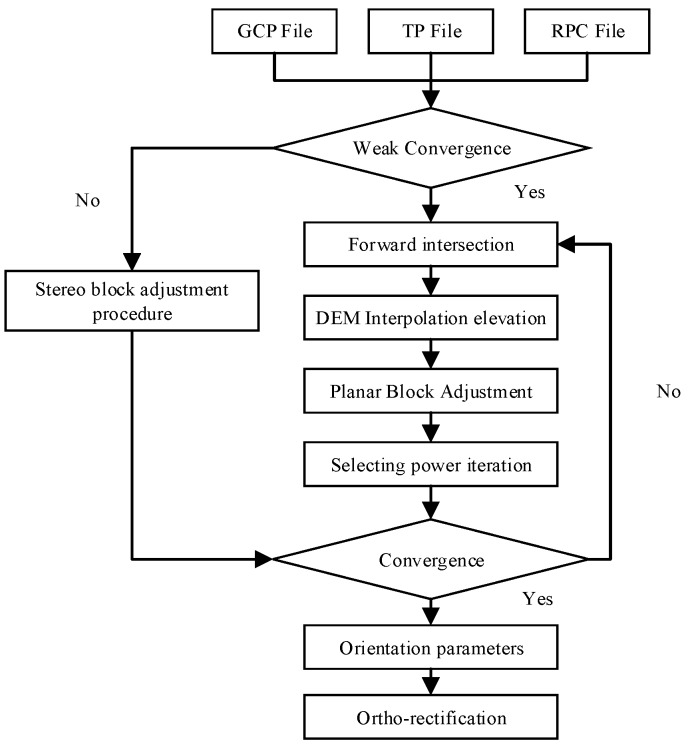
Flowchart of planar block adjustment and ortho-rectification.

Finally, the digital orthophoto map (DOM) of GF-3 image is generated by ortho-rectification. It is a classical method of remote sensing image processing [27]. The image is changed from the image coordinate system to the geodetic coordinate system according to the orientation parameter solved by the block adjustment. The planar block adjustment and orthorectification solution procedure for GF-3 imagery is shown in Figure 3 as follow steps:

(1) Adjustment input file preparation. Including GCP File, TP File, and RPC File. GCP File and TP File are obtained by manual measurement.
(2) Weak convergence determined. Calculating the intersection angle of two SAR images. If the angle is less than 10°, the planar block adjustment process will be executed. Otherwise, the stereo block adjustment will be executed.
(3) Planar block adjustment. First, the initial values of TPs are obtained by forward intersection. Considering the situation of weak intersection. Second, the elevations of TPs are obtained by DEM interpolation. Third, adjustment calculation. Fourth, the posteriori weight method is used to assign weights again. Fifth, if adjustment convergence, it can go to step 4. Otherwise, continue the implementation step 3.
(4) Ortho-rectification.

## 3. Test Results and Analysis

In this study, the test data is the SAR slant range images of GF-3. In order to prove the accuracy of the block adjustment without GCPs for GF-3, we selected two test areas in China. One is Wuhan City in Hubei province and the other is Hubei province. For Wuhan, four-track data including 11 GF-3 images with QPSI model were available, whereas for Hubei, nine-track data including 31 GF-3 images with FS2 model were available. Further details regarding the two test areas are listed in Table 1.

**Figure 4:**
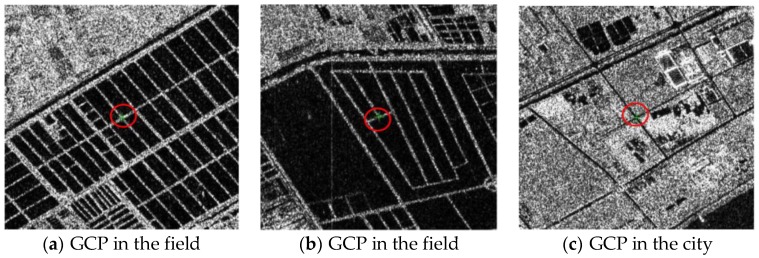
Selection of control/check points in SAR image.

**Figure 5:**
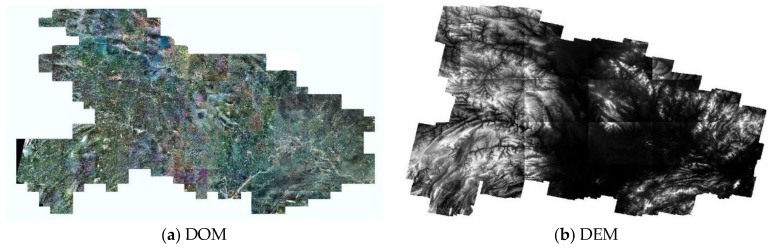
DOM and DEM of Hubei province, China.

Owing to the special imaging modality and the low image signal-to-noise ratios, ground objects in SAR images are more difficult to identify than features in optical images. As it was difficult to measure the GCPs, the annual measurement accuracy of GCPs in both SAR images was about ±1 pixel or even lower. All the GCPs represented prominent ground features such as road intersections or corners of water bodies (Figure 4). The GCPs in Wuhan and Hubei were obtained from DOM and DEM with a spatial accuracy of ±5 m in plane and ±2 m in elevation. The DOM and DEM were generated by ZY-3 satellite stereoscopic surveying and mapping images (Figure 5).

First, to verify the stability of geometric calibration effect of GF-3, a comparative experiment was carried out in the Wuhan test area using GF-3 SAR images before and after calibration.

**Figure 6:**
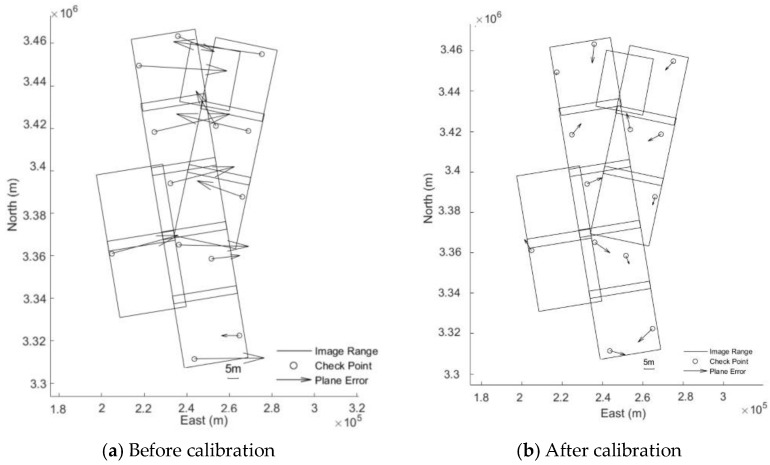
Residual distributions of IPs of block adjustments for Wuhan without GCPs.

From Table 2 and Figure 6, it is easy to see that before and after the geometric calibration, the improvement of the image Independent Check Points’ (ICPs) accuracy of block adjustment is obvious. The ICPs’ accuracy of block adjustment without GCPs before calibration is 29.93 m, while after calibration, it is 7.31 m. Relative to the 8 m resolution of QPSI model, the accuracy is better than 1 pixel in image space.

Meanwhile, the errors of TPs have been calculated before and after calibration, as shown in Table 3. The RMSE of TPs is better than 1 pixel and there is no obvious change before and after the calibration. This shows that the error, which is eliminated by geometric calibration, is obviously systematic, and the relative accuracy between different images of block adjustment will not change due to geometric calibration.

Second, a larger test area of Hubei province is selected. GF-3 SAR images with FS2 model achieved full coverage in Hubei province. Tests, as before, had a comparative block adjustment experiment carried out in Hubei test area using GF-3 SAR images before and after calibration. The accuracy of ICPs and TPs is counted.

**Figure 7:**
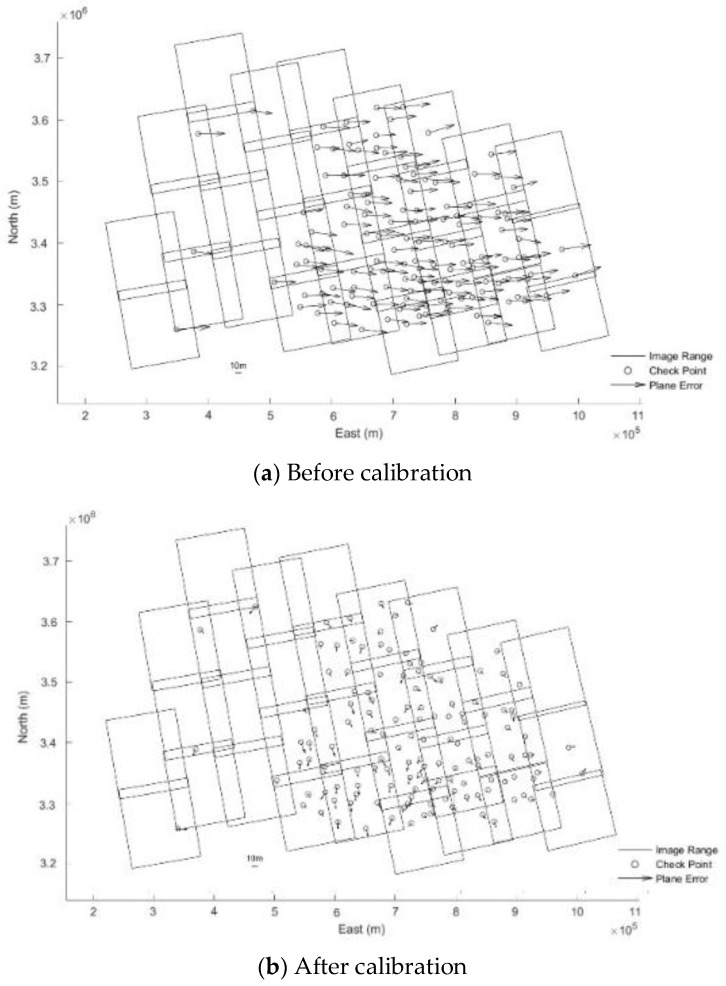
Residual distributions of ICPs of block adjustments for Hubei without GCPs.

**Figure 8:**
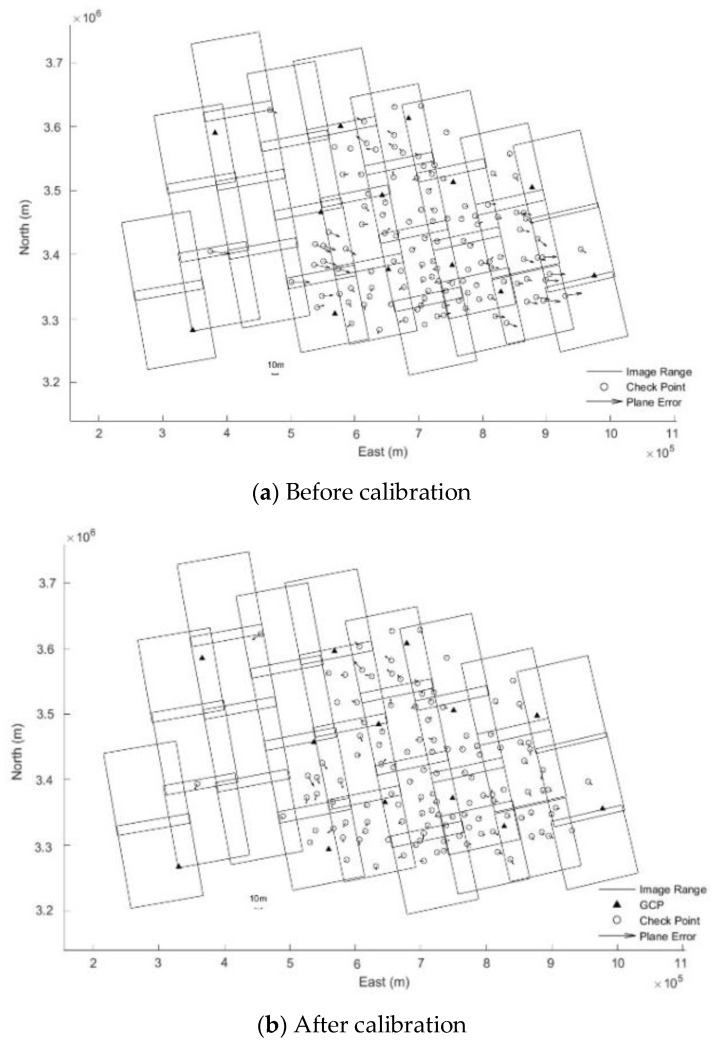
Residual distributions of ICPs of block adjustments for Hubei with GCPs.

After analyzing the results of Table 4 and Table 5, it is found that before and after geometric calibration, the ICPs’ accuracy of block adjustment without GCPs is improved from 38.97 m to 8.97 m. Compared with 10 m resolution of FS2 model, the accuracy is better than one pixel in image space. Additionally, from Figure 7 and Figure 8, it can be seen that residual distributions of ICPs of block adjustments has obvious system aticness for Hubei before geometric calibration. It can be well eliminated by block adjustment after geometric calibration. When a total of 13 GCPs are added (uniform distribution of GCPs’ position in the test area), ICPs’ accuracy of block adjustment before geometric calibration improves less than that after geometric calibration. However, the TPs’ accuracy of block adjustment with and without GCPs before and after geometric calibration does not seem to have changed clearly, which are about one pixel.

To further verify the accuracy of block adjustment after the calibration, test of distribution of different GCPs is added, as shown in Table 6.

From Table 4 and Table 6, the accuracy of ICPs is not significantly improved with the increase of GCP. The accuracy of ICPs is better than eight m when GCPs are added. Compared with the state without GCPs, the accuracy of ICPs has been improved even though geometric calibration is carried out.

**Figure 9:**
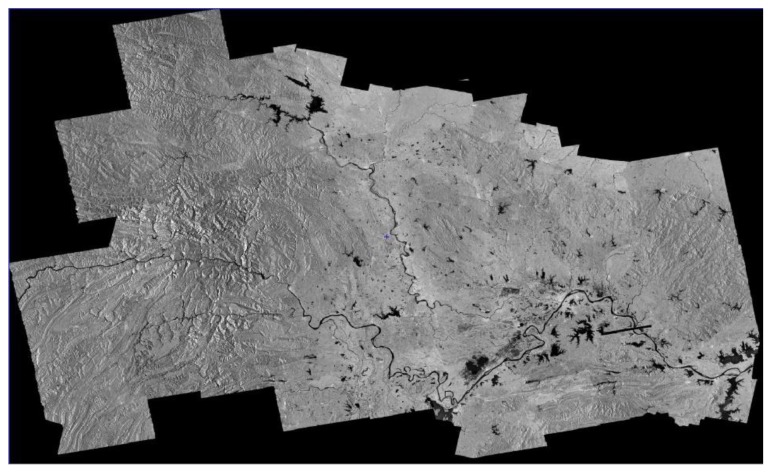
The ortho-map of Hubei province made by GF-3.

**Figure 10:**
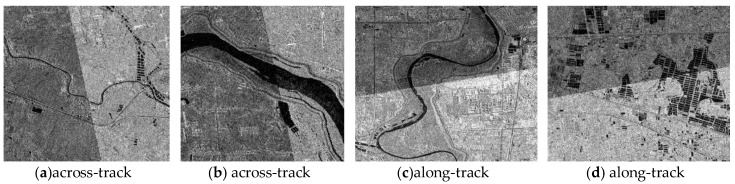
The mosaic maps of adjacent images after ortho-rectification.

Finally, with the orientation parameters of block adjustment for GF-3 after geometric calibration, the ortho-rectification is conducted in Hubei province and the Hubei’s DEM data is introduced to eliminate projection difference caused by terrain undulation [27]. The situation of ortho-map and image mosaic are shown as follows. With Figure 9 and Figure 10, a seamless mosaic result of adjacent image after ortho-rectification shows the high relative geometric accuracy between images, whereas along-track direction or across-track direction.

## 4. Conclusions

In this study, a new method to orthorectify spaceborne SAR images using block adjustment without GCPs was developed and validated using Chinese GF-3 images. The following conclusions can be drawn.

It is feasible to carry out the work of geometric calibration before block adjustment of SAR images. It will eliminate a large part of the systematic error. The proposed method does not require GCPs for orthorectification, reducing the cost for field surveys, especially for images covering large areas. In addition, it can guarantee consistent mosaic accuracy when producing orthoimages of an entire area. In the process, an almost complete geometric seamless mosaic orthoimage is achieved, making the proposed method a good foundation for producing image mosaics. By conducting block adjustment without GCPs on GF-3 data from two different areas, a horizontal accuracy of ICPs better than one pixel can be achieved and the accuracy of TPs can also achieve the level better than one pixel. This method can lay a good geometric foundation for global mapping with GF-3 images.

## Figures and Tables

**Table 1:** Basic parameters of the test area.

Item	Wuhan City	Hubei Province
Imaging mode	QPSI	FS2
Nominal resolution (m)	8	10
Width of image (km)	30	100
Number of orbit	4	9
Number of images	11	31
Number of GCPs/ICPs	8	134
Number of tie points	33	1038
terrain	plain	Mountains, hills, plain
area (km^2^)	8594	185,900

ICPs: Independent Check Points, playing a role in checking the accuracy of adjustment.

**Table 2:** ICPs’ accuracy of block adjustment without GCPs using GF-3 SAR images before and after calibration.

Test Area	Scheme	GCP	ICP	Maximum Error (m)			RMSE (m)		
				x	y	plane	x	y	plane
Wuhan	before calibration	0	13	−41.50	16.55	41.91	29.03	7.31	29.93
	after calibration	0	13	7.75	−9.45	9.73	4.99	5.34	7.31

**Table 3:** TPs’ accuracy of block adjustment for Wuhan without GCPs before and after calibration.

Test Area	Scheme	TP	Maximum Error (pixel)			RMSE (pixel)		
			x	y	plane	x	y	plane
Wuhan	before calibration	33	0.96	−0.95	0.96	0.35	0.23	0.42
	after calibration	33	0.86	−0.98	0.99	0.34	0.23	0.41

**Table 4:** ICPs’ accuracy of block adjustment for Hubei without GCPs before and after calibration.

Test Area	Scheme	GCP	ICP	Maximum Error (m)			RMSE (m)		
				x	y	plane	x	y	plane
Hubei	before calibration	0	135	54.80	13.74	55.01	38.70	4.54	38.97
		13	122	32.32	−13.95	32.63	11.35	4.88	12.35
	after calibration	0	135	14.90	−17.98	19.10	4.60	7.70	8.97
		13	122	−13.10	−16.51	18.81	4.82	5.42	7.26

**Table 5:** TPs’ accuracy of block adjustment for Hubei without GCPs before and after calibration.

Test Area	Scheme	GCP	TP	Maximum Error (pixel)			RMSE (pixel)		
				x	y	plane	x	y	plane
Hubei	before calibration	0	1038	−2.72	2.65	2.92	0.67	0.61	0.91
		13	1038	−5.05	−2.80	5.05	0.95	0.65	1.15
	after calibration	0	1038	3.12	2.66	3.13	0.66	0.61	0.90
		13	1038	3.11	−2.79	3.11	0.70	0.65	0.95

**Table 6:** ICPs’ accuracy of block adjustment for Hubei with different GCPs after calibration.

Test Area	Scheme	GCP	Maximum Error (m)			RMSE (m)		
			x	y	plane	x	y	plane
Hubei	After calibration	1	15.00	−17.42	18.41	4.53	6.55	7.96
		4	10.93	−17.14	18.12	4.38	5.50	7.03
		9	−12.74	−16.27	18.39	4.59	5.50	7.17
		13	−13.10	−16.51	18.81	4.82	5.42	7.26
